# Use of aromatase inhibitors in patients with breast cancer is associated with deterioration of bone microarchitecture and density

**DOI:** 10.20945/2359-3997000000385

**Published:** 2021-07-16

**Authors:** Frederico Arthur Pereira Nunes, Maria Lucia Fleiuss de Farias, Felipe Peres Oliveira, Leonardo Vieira, Luis Felipe Cardoso Lima, Francisco de Paula Paranhos, Laura Maria Carvalho de Mendonça, Miguel Madeira

**Affiliations:** 1 Universidade Federal do Rio de Janeiro Divisão de Endocrinologia Rio de Janeiro RJ Brasil Divisão de Endocrinologia, Universidade Federal do Rio de Janeiro, Rio de Janeiro, RJ, Brasil; 2 Hospital Federal Cardoso Fontes Departamento de Oncologia Rio de Janeiro RJ Brasil Departamento de Oncologia, Hospital Federal Cardoso Fontes, Rio de Janeiro, RJ, Brasil; 3 Universidade Federal do Rio de Janeiro Programa de Engenharia Nuclear Rio de Janeiro RJ Brasil Programa de Engenharia Nuclear, Universidade Federal do Rio de Janeiro, Rio de Janeiro, RJ, Brasil; 4 Universidade Federal do Rio de Janeiro Divisão de Reumatologia Rio de Janeiro RJ Brasil Divisão de Reumatologia, Universidade Federal do Rio de Janeiro, Rio de Janeiro, RJ, Brasil

**Keywords:** Breast cancer, osteoporosis, aromatase inhibitors (AI), bone mineral density (BMD), high resolution peripheral quantitative computed tomography (HR-pQCT)

## Abstract

**Objective:**

To evaluate changes in bone density and architecture in postmenopausal women with breast cancer (BC) and use of aromatase inhibitor (AI).

**Subjects and methods:**

Thirty-four postmenopausal women with BC, without bone metastasis, renal function impairment and who were not receiving bone-active drugs were selected from a population of 523 outpatients treated for BC. According to the presence of hormonal receptors, HER2 and Ki67, seventeen had positive hormonal receptors and received anastrozole (AI group), and seventeen were triple-negative receptors (non-AI group), previously treated with chemotherapy. Areal bone mineral density (aBMD) and vertebral fracture assessment (VFA) analyses were performed by DXA; vBMD and bone microarchitecture were evaluated by HR-pQCT. Fracture risk was estimated using the FRAX tool.

**Results:**

No patient referred previous low-impact fracture, and VFA detected one moderate vertebral fracture in a non-AI patient. AI patients showed lower aBMD and BMD T-scores at the hip and 33% radius and a higher proportion of osteoporosis diagnosis on DXA (47%) vs non-AI (17.6%). AI group had significantly lower values for vBMD at the entire, cortical and trabecular bone compartments, cortical and trabecular thickness and BV/TV. They also had a higher risk for major fractures and for hip fractures estimated by FRAX. Several HR-pQCT parameters evaluated at distal radius and distal tibia were significantly associated with fracture risk.

**Conclusion:**

AI is associated with alterations in bone density and microarchitecture of both the cortical and trabecular compartments. These findings explain the overall increase in fracture risk in this specific population.

## INTRODUCTION

Breast cancer (BC) is the most prevalent cancer type for women. Approximately 1.67 million new cases of BC were diagnosed in 2012 worldwide, representing 25% of all cancers in women. For Brazil, in 2018, 59,700 new cases of BC were expected ( 1 ). In 2019, the expectation is for 268,600 new cases and 41,760 deaths caused by this disease in the USA ( 2 ).

The presence of hormone receptors expressed by the tumor cells guides treatment. Approximately 70% of patients with BC have tumors with positive hormone receptors (R+), and those with potentially curable disease benefit from adjuvant hormone therapy ( 3 ).

In postmenopausal (PM) women, estrogen synthesis depends mostly on the conversion of adrenal precursors by the enzyme aromatase, which is present in extragonadal sites, mainly adipose tissue ( 4 ). After menopause, a negative imbalance in bone remodeling is expected, with accelerated bone loss, especially in the first 15-20 years. Approximately 52% to 66% of this loss occurs due to estrogen deficiency, and the rest stems from aging ( 5 ). Aromatase inhibitors (AIs) block aromatase activity, making circulating levels of PM estrogen virtually undetectable. Several studies have shown the oncological benefit of AI treatment in PM women with (R+BC). Estrogenic activity suppression increases the bone loss rate to approximately 2.6% per year and favors fragility fractures ( 4 , 6 , 7 ).

In the USA, the economic impact of osteoporosis on the health care system is estimated to reach $ 25.3 billion per year by 2025 ( 8 ). In Brazil, there are currently 121,000 hip fractures per year, and projections are that numbers will rise to 140,000 in 2020 with enormous costs (about R$ 1.2 billion per year) ( 9 ). The only way of changing this picture is identify people at great risk and start antiosteoporosis treatment. The clinical evaluation of bone health is based on measures of areal bone mineral density (BMD) using dual X-ray absorptiometry (DXA) ( 10 ). A history of previous low-impact fractures or detection of non-clinical vertebral fractures on X-rays or during DXA exams (vertebral fracture assessment – VFA) also confirm osteoporosis. The Fracture Risk Assessment Tool (FRAX) is recommended by the World Health Organization to estimate the 10-year risk for hip and major fractures, and it was normalized in 2013 for the Brazilian population ( 9 – 11 ).

However, a considerable proportion of PM women and elderly men develop fragility fractures despite not having an osteoporosis diagnosis by DXA ( 12 , 13 ). In fact, BMD accounts for only 70%-75% of the variation in bone strength, while other factors (macro and microarchitecture, tissue composition and microcrack accumulation) correspond to the remainder of this variation. Within this context, the concept of bone quality has gained importance. Both bone compartments (cortical or trabecular) contribute to bone resistance, and they are differentially affected by age, gender, comorbidities and treatments ( 14 , 15 ). Three-dimensional high-resolution peripheral quantitative computed tomography (HR-pQCT) allows for evaluations of volumetric BMD and microstructure at the trabecular and cortical compartments, separately. This allows for a better understanding of alterations in bone geometry and strength associated with increased fracture risk ( 16 ).

This study aimed to evaluate the impact of AI on bone health of PM women with BC, based on the detection of previous fractures, estimation of fracture risk using FRAX, bone density and microarchitecture evaluated by DXA and HR-pQCT.

## SUBJECTS AND METHODS

### Subjects

This was a single-center, cross-sectional and observational study of PM women with BC admitted to Cardoso Fontes Federal Hospital (Rio de Janeiro, Brazil) (Ethics committee approval number: 64781417.0.0000.8066; Ethics committee's feedback number: 2.015.685). The BC diagnoses were based on histopathology. Specifically, immunohistochemistry evaluations were utilized to determine hormonal receptor and HER2 expression as well as Ki67 status in BC cells. Patients younger than 75 years on the second to fifth year of adjuvant AI therapy were eligible to participate in this research protocol. Age-matched patients with BC considered negative for hormonal receptors comprised the non-AI group. Clinical data were collected from medical records, and patients were interviewed about previous low-impact fractures. Every patient submitted to chemotherapy had discontinued this treatment for at least 2 years prior to entrance into this study. The exclusion criteria were as follows: weight above 120 kg (limitations of the densitometer), metastatic disease, other preexisting bone diseases (such as Paget's disease, hyperparathyroidism and hypoparathyroidism), renal failure, prednisone use ≥5 mg for 3 months or more) and use of anti-osteoporosis medications (e.g., bisphosphonates, denosumab and teriparatide).

The protocol was approved by the Research Ethics Committee of the hospital, and all patients received and signed the informed consent form before participating in the protocol, which was in accordance with the Second Declaration of Helsinki.

### FRAX

The FRAX tool, adjusted for the Brazilian population, was employed to estimate a 10-year probability of hip and major fractures ( 9 ). All participants were considered to have secondary causes of osteoporosis.

### Areal bone density and VFA

A Prodigy densitometer (GE Lunar Prodigy Advance, GE Healthcare, Madison, WI, USA) was used for DXA assessment of areal BMD at the lumbar spine, femoral neck, total femur and 33% radius, and the results were expressed as absolute values (g/cm^2^) and standard deviations (SDs) from the expected BMD for young women (T-score). According to the ISCD criteria ( 10 ), patients were identified as having low bone density (previously referred to as osteopenia) or osteoporosis when the lowest BMD T-score was between < −1 and > −2.5 SD or ≤ −2.5 SD, respectively. Patients with a BMD Z-score ≤ −2 SD at any site were considered as having a lower than expected BMD for their age. The variability coefficients of the BMD values were estimated at 1.5% at the lumbar spine and 2.3% at the hip. VFA was also performed during the DXA examinations. The same accredited technician analyzed all images.

### HR-pQCT

Volumetric BMD (vBMD) and bone microarchitecture were measured on the appropriately immobilized non-dominant distal forearm and tibia using a 3D HR-pQCT system (Xtreme CT, SCANCO Medical AG, Brüttisellen, Switzerland). This system employs a 2D detector combined with a 0.08-mm point-focus X-ray tube, which enables the acquisition of several CT sections with an 82-µm nominal resolution. A total of 110 sections were obtained at each site, generating a 9-mm 3D representation in the axial direction. The radiation dose was similar to that used in standard DXA procedures (less than 3 µSv per measurement). The attenuation data were transformed to equivalent hydroxyapatite (HA) densities. Additional details of image acquisition and analysis have been described previously ( 17 ). The variables included in the analysis were as follows: volumetric BMD (g HA/cm^3^) in the trabecular (Dtrab), cortical (Dcomp) or total (Dtotal) region; cortical thickness (CTh, mm); fraction of trabecular bone volume to tissue volume (BV/TV); trabecular thickness (TbTh, mm); trabecular number (TbN, mm^−1^); trabecular separation (TbSp, mm); and standard deviation of the TbSp (TbSp 1/N SD, mm), which reflects the heterogeneity of the trabecular network. TbTh and TbSp were calculated based on the TbN and BV/TV [TbTh = BV/TV/TbN, and TbSp (1-BV/TV)/TbN]. CTh was calculated by dividing the cortical volume by the external bone surface area. The variability of density-based measurements was less than 1% and between 3% and 5% for the bone structural parameters ( 18 , 19 ).

### Statistics

The statistical analyses were performed using SPSS version 20.0 for MacOS (SPSS Inc., Chicago, IL, USA). In the descriptive analysis, the categorical variables were expressed through their percentages and frequencies. Numerical variables with a normal distribution were expressed as the mean ± standard deviation, while the variables with asymmetric distributions were expressed with medians (minimum - maximum). The Kolmogorov-Smirnov test was performed to evaluate the distribution pattern of the numerical variables. The Student's T-test or the Mann-Whitney U-test were performed to compare numerical variables between the two groups, as appropriate. The chi-squared or Fisher's exact tests were applied to compare categorical variables, as appropriate. Correlations between the numerical variables were analyzed using the Spearman correlation test. Bi-caudate tests were used in all analyzes. The limit of statistical significance was 5%.

## RESULTS

**Figure 1:**
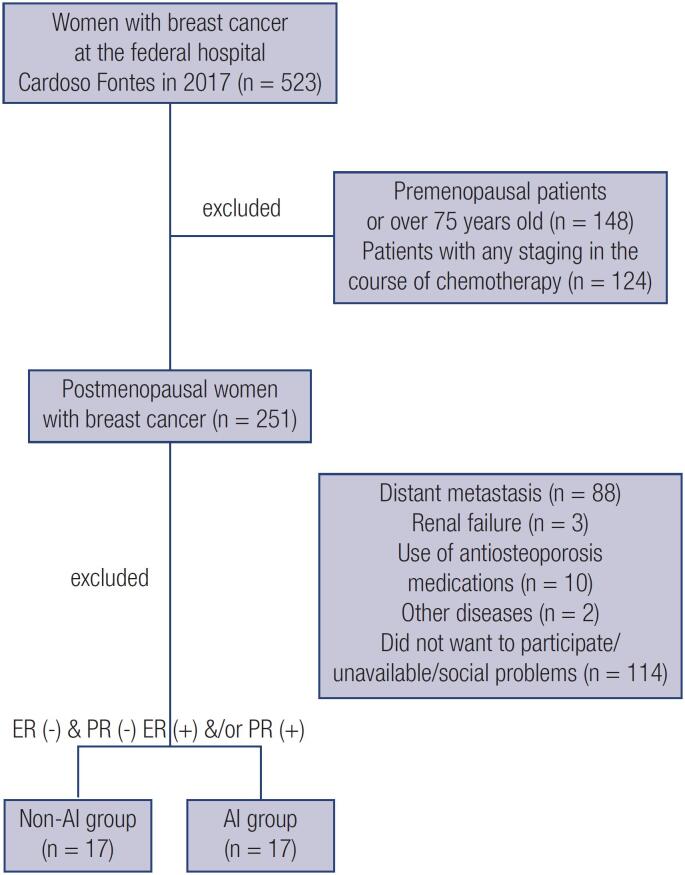
Flowchart of study subjects. A total of 251 postmenopausal women with breast cancer were pre-selected and 34 included. ER: estrogen receptor; PR: progesterone receptor; AI: aromatase inhibitor.

A total of 34 patients participated in the study according to the criteria mentioned in Figure 1 . Seventeen patients were assigned to the AI group and 17 patients to the non-AI group. The AI patients received letrozole or anastrozole for 2-5 years (mean 3.11 ± 1.00 years), six of them used tamoxifen and 9 had been on chemotherapy prior to the AI treatment. All receptor negative patients received chemotherapy. No difference was found between the AI and non-AI groups concerning age at the study (62.00 ± 5.80 *vs.* 57.05 ± 8.96 years, p = 0.066), age at menopause (47.94 ± 6.24 *vs.* 46.35 ± 4.74 years, p = 0.410), time elapsed since menopause (13.47 ± 8.24 *vs.* 10.76 ± 9.79 years, p = 0.390), the number of patients referring regular physical exercise (10 *vs.* 8), type 2 diabetes mellitus (6 *vs.* 5), or ethnicity. The AI patients had lower body mass indexes (26.55 ± 3.22 *vs.* 30.88 ± 7.10 kg/m^2^, p = 0.034).

There was no history of previous low-impact fracture in either group and VFA detected only one morphometric fracture in a non-AI patient (moderate fracture of T6). However, the FRAX-Brazil tool estimated a higher risk for major (AI 3.47% ± 1.57 *vs.* non-AI 2.50% ± 0.76, p = 0.029) and hip fractures (AI 0.50% [0-4.6] *vs.* non-AI 0.20% [0-1.9], p = 0.010).

Bone densitometry revealed decreased BMD and T-scores in the hips and 33% radius ( Table 1 ). Furthermore, the distribution of patients classified as having normal BMD (2 AI *vs.* 9 non-AI), low BMD (7 AI *vs.* 5 non-AI) and osteoporosis (8 AI *vs.* 3 non-AI) was significantly different between the groups (p = 0.029). Additionally, 5 AI patients and only 1 non-AI patient had lower than expected BMD for their age. The HR-pQCT confirmed alterations in bone density and differences in the bone microarchitecture between the groups ( Table 1 ).

**Table 1:** Bone densitometry and high-resolution peripheral quantitative computed tomography data of patients with breast cancer receiving aromatase inhibitors (AI) or not (non-AI)

		AI (n = 17)	Non-AI (n = 17)	p-value
DXA				
	LS BMD (g/cm^2^)	1.030 ± 0.159	1.121 ± 0.165	0.127
	FN BMD (g/cm^2^)	0.901 ± 0.081	0.992 ± 0.123	**0.018**
	TF BMD (g/cm^2^)	0.910 ± 0.098	1.013 ± 0.127	**0.014**
	33%R BMD (g/cm^2^)	0.583 ± 0.073	0.694 ± 0.064	**<0.001**
HR-pQCT				
radius				
	D100 (mgHA/cm^3^)	272.01 ± 41.23	341.58 ± 61.51	**0.001**
	D comp (mgHA/cm^3^)	836.80 ± 52.31	890.70 ± 57.60	**0.009**
	Ct. Th (mm)	0.64 ± 0.12	0.80 ± 0.16	**0.003**
	D trab (mgHA/cm^3^)	124.01 ± 29.36	163.35 ± 37.88	**0.002**
	BV/TV	0.10 ± 0.02	0.14 ± 0.03	**0.003**
	Tb.N (mm^−1^)	1.75 ± 0.32	1.95 ± 0.26	0.055
	Tb.Th (mm)	0.06 ± 0.01	0.07 ± 0.01	**0.007**
	Tb. Sp (mm)	0.54 ± 0.12	0.45 ± 0.08	**0.032**
	Tb.1/N.SD (mm)	0.26 ± 0.11	0.20 ± 0.05	0.098
tibia				
	D100 (mgHA/cm^3^)	261.50 ± 44.10	296.44 ± 66.10	0.088
	D comp (mgHA/cm^3^)	833.90 ± 59.40	884.47 ± 61.21	**0.024**
	Ct. Th (mm)	1.07 ± 0.19	1.17 ± 0.28	0.233
	D trab (mgHA/cm^3^)	130.74 ± 30,56	153.31 ± 38.03	0.074
	BV/TV	0.11 ± 0.03	0.13 ± 0.03	0.073
	Tb.N (mm^−1^)	1.55 ± 0.31	1.70 ± 0.30	0.158
	Tb.Th (mm)	0.07 ± 0.01	0.07 ± 0.01	0.327
	Tb. Sp (mm)	0.61 ± 0.17	0.53 ± 0.09	0.111
	Tb.1/N.SD (mm)	0.35 ± 0.20	0.25 ± 0.06	0.088

LS: lumbar spine; FN: femoral neck; TF: total femur; 33%R: radius 33%; D100: vBMD of entire bone; D comp: cortical vBMD; Ct. Th: cortical thickness; D trab: trabecular vBMD; BV/TV: bone volume/total volume ratio; Tb. N: trabecular number; Tb. Th: trabecular thickness; Tb. Sp: trabecular separation; Tb.1/N.SD: inhomogeneity of trabecular network.

The only microstructural parameter significantly associated with age was Dcomp, at the distal radius (r −0.379, p = 0.027) and at the distal tibia (r −0.454, p = 0.007). BMI was positively associated with Ct. Th at the radius (r 0.352, p = 0.041) and tibia (r 0.367, p = 0.033) as well as with Dcomp (r 0.367, p = 0.033) and TbN (r 0.364, p = 0.034), both at the distal tibia.

Fracture risk estimated using the FRAX tool was also negatively influenced by lumbar spine BMD (r −0.456, p = 0.008 for major fractures and r −0.455, p = 0.008 for hip fractures) and bone microstructure, including trabecular and cortical indexes, as shown in Table 2 .

**Table 2:** Correlations between microstructural parameters and fracture risk estimated by FRAX

		FRAX Major	fracture	FRAX Hip fracture	
		r	p-value	r	p-value
Radius					
	D100	−0.499	0.003	−0.487	0.004
	D comp	−0.573	<0.001	−0.551	0.001
	Ct. Th	−0.587	<0.001	−0.561	0.001
	D trab	−0.388	0.023	−0.387	0.024
	BV/TV	−0.390	0.023	−0.390	0.023
Tibia					
	D100	−0.631	<0.001	−0.365	<0.001
	D comp	−0.709	<0.001	−0.698	<0.001
	Ct. Th	−0.544	0.001	−0.540	0.001
	D trab	−0.513	0.002	−0.524	0.001
	BV/TV	−0.517	0.002	−0.527	0.001
	Tb.N	−0.456	0.007	−0.426	0.012
	Tb.1/N.SD	0.477	0.004	0.468	0.005

## DISCUSSION

This study demonstrates that AIs used in BC patients is associated with higher risk of fragility fractures (evaluated by FRAX) and decreased bone density and quality (assessed by DXA and HR-pQCT) with negative effect on both trabecular and cortical bones.

Several studies have evaluated the effect of AIs on areal bone density as well as its therapeutic possibilities ( 20 ). In the ATAC study, for example, routine BMD monitoring showed that the highest rate of bone loss occurs in the first two years of AI use. There was a decrease in lumbar spine and hip BMD levels in the AI patients (6.08% and 7.24%, respectively) in contrast with those on tamoxifen, who showed a BMD gain of 2.88% and 0.74%, respectively. AI discontinuation led to a BMD increase at the lumbar spine and no further hip loss ( 21 ).

A better understanding of bone properties can be obtained by the histomorphometry analysis of bone biopsy, which is an invasive and expensive method. QCT and HR-pQCT for bone study indirectly assess these parameters and help to clarify the changes associated with bone fragility.

There is a growing number of clinical trials using QCT and HR-pQCT for evaluation of bone quality; however, only a few have evaluated BC patients. Lee and cols.( 22 ) utilized QCT in the lumbar spine and femur to study the influence of AIs on bone. Like our DXA data, they did not find differences in lumbar spine BMD, but vBMD was decreased in the femoral neck and total femur in their AI patients. The cortical bone compartment was especially affected in their AI patients, which is in accordance with our findings of deleterious effects on the cortical bone. The authors also reported that bone loss in their AI patients was negatively related to age and time on AI treatment and was positively associated with BMI. The authors concluded that AI treatment was associated with deterioration of femoral cortical BMD and geometry, which could contribute to site-specific decreased bone strength and increased incidence of hip fractures ( 22 ).

Szabo and cols. ( 23 ) utilized peripheral QCT (pQCT) to compare BC patients on AI treatment and healthy PM women. Their AI patients demonstrated significantly lower total vBMD values (4% at radius and at tibia) and lower cortical densities (20% at radius and 38% at tibia). We found similar data, such as significant decreases in total bone, cortical and trabecular vBMDs, but the HR-pQCT could also detect decreased trabecular and cortical thickness as well as reduced trabecular BV/TV.

Only one study utilized HR-pQCT and DXA in patients with BC, but in patients receiving AI drug (exemestane) for 2 years ( 24 ). There was a significant decline in aBMD (DXA) in the lumbar spine, femoral neck and total femur, as well as in the total, cortical and trabecular vBMD and cortical thickness (radius and tibia) and BV/TV in the distal radius. We also found similar alterations in both compartments but mainly in the cortical bone in patients receiving anastrozole or letrozole.

There are several tools for evaluating fracture risk related to osteoporosis – FRAX being the most utilized. It contemplates several risk factors, including secondary causes of osteoporosis, such as the use of drugs interfering with bone health, although not specifically AIs ( 11 ). Mariotti and cols. ( 25 ) showed that the combination of FRAX, trabecular bone score and BMD maximized the identification of BC patients with elevated fracture risk. Cheung and cols. ( 24 ) evaluated fracture risk based on the FRAX tool in patients receiving exemestane but could not conclude that a decline occurred in their 2-year study period.

In this study, the group receiving AI had a higher FRAX fracture risk for major and hip fractures. We searched for previous fractures in the patient histories as well as for non-clinical vertebral fractures by VFA, and we believe that the absence of fractures might be due to their short times on AIs and the small sample size (type 2 error). The lower BMI in the AI patients may have contributed to alterations in bone microstructure at the distal tibia, which is a weight bearing bone. The most important finding was the significant correlation of fracture risk with bone microarchitecture, both in the trabecular and cortical compartments, which was undoubtedly deranged in the AI patients.

Our study has limitations due to its cross-sectional design and the small sample size. However, we detected a clear deterioration in bone density and microarchitecture in both cortical and trabecular bone using HR-pQCT, which might explain the bone fragility and increased fracture risk in the BC patients receiving AIs. An important information regarding the two subgroups is that the patients in the control group (triple negative) had higher body weight, which may give this group a protective factor against bone loss. However, also in this group, all patients received chemotherapy, which can lead to loss of bone mass. Another relevant data is the use of the subtypes of AIs. Some studies have suggested a less significant effect of exemestane on bone loss, due to its androgenic structure, compared to letrozole, while other clinical studies comparing different AIs did not reveal any significant difference ( 26 ). In our study, due to standardized medication in the hospital, anastrozole was basically used as a drug in the adjuvant treatment.

We are facing a public health problem that affects many people worldwide: BC and osteoporosis. Our study reinforces the importance of assessing bone health early in women diagnosed with BC, with PM status who will use adjuvant therapy with AIs. Early diagnosis can thus prevent bone events, financial cost in their treatments and worsening quality of life.

The use of HR-pQCT adds valuable information to the understanding of women's bone health and fracture risk. Future studies with a larger population of BC patients receiving AIs may deepen the understanding of how bone is impacted by this treatment modality.

## References

[1] Brazilian National Cancer Institute (Inca).

[2] National Cancer Institute Surveillance, Epidemiology, and End Results Program.

[3] Bae SY, Kim S, Lee JH, Lee HC, Lee SK, Kil WH (2015). Poor prognosis of single hormone receptor-positive breast cancer: similar outcome as triple-negative breast cancer. BMC Cancer.

[4] Mackey JR, Joy AA (2005). Skeletal health in postmenopausal survivors of early breast cancer. Int J Cancer.

[5] Erbag G, Uygun K, Binnetoglu E, Korkmaz AN, Asik M, Sen H (2015). Aromatase inhibitor treatment for breast cancer: short-term effect on bone health. Contemp Oncol (Pozn).

[6] Chumsri S (2015). Clinical utilities of aromatase inhibitors in breast cancer. Int J Womens Health.

[7] Perez EA, Serene M, Durling FC, Weilbaecher K (2006). Aromatase inhibitors and bone loss. Oncology (Williston Park).

[8] Qaseem A, Forciea MA, McLean RM, Denberg TD (2017). Treatment of low bone density or osteoporosis to prevent fractures in men and women: A clinical practice guideline update from the American College of Physicians. Ann Intern Med.

[9] Aziziyeh R, Amin M, Habib M, Garcia Perlaza J, Szafranski K, McTavish RK (2019). The burden of osteoporosis in four Latin American countries: Brazil, Mexico, Colombia, and Argentina. J Med Econ.

[10] Silva BC, Broy SB, Boutroy S, Schousboe JT, Shepherd JA, Leslie WD (2015). Fracture Risk Prediction by Non-BMD DXA Measures: the 2015 ISCD Official Positions Part 2: Trabecular Bone Score. J Clin Densitom.

[11] Kanis JA, Johnell O, Oden A, Johansson H, McCloskey E (2007). FRAX and the assessment of fracture probability in men and women from the UK. Osteoporos Int.

[12] Sornay-Rendu E, Munoz F, Duboeuf F, Delmas P (2005). Rate of forearm bone loss is associated with an increased risk of fracture independently of bone mass in postmenopausal women: the OFELY study. J Bone Miner Res.

[13] Schuit SC, van der Klift M, Weel AE, de Laet CE, Burger H, Seeman E (2004). Fracture incidence and association with bone mineral density in elderly men and women: the Rotterdam Study. Bone.

[14] Ammann P, Rizzoli R (2003). Bone strength and its determinants. Osteoporos Int.

[15] Seeman E, Delmas PD (2006). Bone quality – the material and structural basis of bone strength and fragility. N Engl J Med.

[16] Burghardt AJ, Link TM, Majumdar S (2011). High-resolution computed tomography for clinical imaging of bone microarchitecture. Clin Orthop Relat Res.

[17] Laib A, Hauselmann HJ, Ruegsegger P (1998). In vivo high-resolution 3D-QCT of the human forearm. Technol Health Care.

[18] Boutroy S, Bouxsein ML, Munoz F, Delmas PD (2005). In vivo assessment of trabecular bone microarchitecture by high-resolution peripheral quantitative computed tomography. J Clin Endocrinol Metab.

[19] Griffith JF, Engelke K, Genant H (2010). Looking beyond bone mineral density imaging assessment of bone quality. Ann N Y Acad Sci.

[20] Body J.J (2012). Aromatase inhibitors-induced bone loss in early breast cancer. Bonekey Rep.

[21] Eastell R, Adams JE, Coleman RE, Howell A, Hannon RA, Cuzick J (2008). Effect of Anastrozole on Bone Mineral Density: 5-Year Results From the Anastrozole, Tamoxifen, Alone or in Combination Trial 18233230. J Clin Oncol.

[22] Lee SJ, Kim KM, Brown JK, Brett A, Roh YH, Kang DR (2015). Negative impact of aromatase inhibitors on proximal femoral bone mass and geometry in postmenopausal women with breast cancer. Calcif Tissue Int.

[23] Szabo KA, Webber CE, Adachi JD, Tozer R, Gordon C, Papaioannou A (2011). Cortical and trabecular bone at the radius and tibia in postmenopausal breast cancer patients: A peripheral quantitative computed tomography (pQCT) study. Bone.

[24] Cheung AM, Tile L, Cardew S, Pruthi S, Robbins J, Tomlinson G (2012). Bone density and structure in healthy postmenopausal women treated with Exemestane for the primary prevention of breast cancer: A nested substudy of the MAP.3 randomised controlled trial. Lancet Oncol.

[25] Mariotti V, Page DB, Davydov O, Hans D, Hudis CA, Patil S (2016). Assessing fracture risk in early stage breast cancer patients treated with aromatase-inhibitors: An enhanced screening approach incorporating trabecular bone score. J Bone Oncol.

[26] Smith I, Yardley D, Burris H, De Boer R, Amadori D, McIntyre K (2017). Comparative efficacy and safety of adjuvant letrozole versus anastrozole in postmenopausal patients with hormone receptor-positive, node-positive early breast cancer: final results of the randomized phase III femara versus anastrozole clinical evaluation (FACE) trial. J Clin Oncol.

